# Supplementary material for: Saccharomyces boulardii (CNCM I-745) improves intestinal damage in sepsis by remodeling intestinal flora structure
Source: Front Cell Infect Microbiol. 2025 Jul 31;15:1602792. doi: 10.3389/fcimb.2025.1602792 (PMC12350329; doi:10.3389/fcimb.2025.1602792)
Supplement: Supplementary file 1 [file DataSheet1.zip › summary/summary/6_Taxonomy_community/1_Taxonomy_profiling/3_Class/Group/Class_abund_top30_Group.heatmap.pdf]

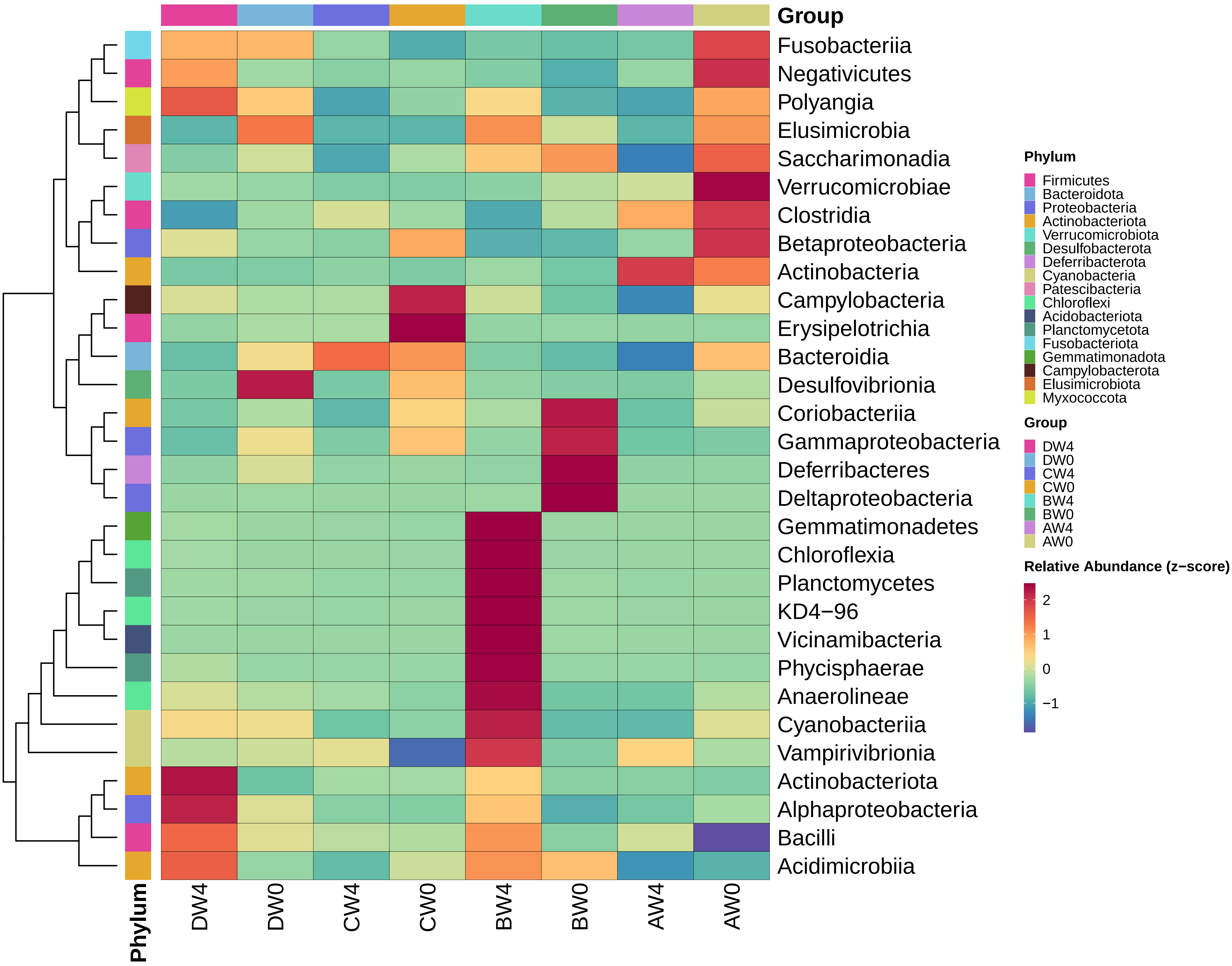

Group
Fusobacteriia
Negativicutes
Polyangia
Elusimicrobia
Saccharimonadia
Verrucomicrobiae
Clostridia
Betaproteobacteria
Actinobacteria
Campylobacteria
Erysipelotrichia
Bacteroidia
Desulfovibrionia
Coriobacteriia
Gammaproteobacteria
Deferribacteres
Deltaproteobacteria
Gemmatimonadetes
Chloroflexia
Planctomycetes
KD4−96
Vicinamibacteria
Phycisphaerae
Anaerolineae
Cyanobacteriia
Vampirivibrionia
Actinobacteriota
Alphaproteobacteria
Bacilli
Acidimicrobiia
Phylum
DW4
DW0
CW4
CW0
BW4
BW0
AW4
AW0
Phylum
Firmicutes
Bacteroidota
Proteobacteria
Actinobacteriota
Verrucomicrobiota
Desulfobacterota
Deferribacterota
Cyanobacteria
Patescibacteria
Chloroflexi
Acidobacteriota
Planctomycetota
Fusobacteriota
Gemmatimonadota
Campylobacterota
Elusimicrobiota
Myxococcota
Group
DW4
DW0
CW4
CW0
BW4
BW0
AW4
AW0
Relative Abundance (z−score)
2
1
0
−1
